# Cognitive behavioral therapy for postpartum panic disorder: a case series

**DOI:** 10.1186/s40359-019-0330-z

**Published:** 2019-08-22

**Authors:** Kazuki Matsumoto, Koichi Sato, Sayo Hamatani, Yukihiko Shirayama, Eiji Shimizu

**Affiliations:** 1 Research Center for Child mental Development, Chiba University, Chiba, Japan; 2 Department of Psychiatry, Teikyo University Chiba Medical Center, 3426-3, Anegasaki, Ichihara-shi, Chiba, Japan; 3 Research Fellow of japan Society for the Promotion of Science, Chiba, Japan; 4 Department of Cogntiive Behavioral Physiology, Guraduate School of Medicine, Chiba University, Chiba, Japan

**Keywords:** Postpartum panic disorder, Agoraphobia, Cognitive behavioral therapy

## Abstract

**Background:**

Clinical anxiety is common during the perinatal period, and anxiety symptoms often persist after childbirth. Ten to 30 % of perinatal women are diagnosed with panic disorder (PD)—far more than the 1.5–3% rate among the general population. Although cognitive behavioral therapy (CBT) has been determined to be an effective treatment for PD, few studies have been conducted on CBT effectiveness in treating postpartum PD and, to the best of the knowledge of the present authors, no research has been conducted on postpartum PD among Japanese women. In this manuscript, we report on our administration of CBT to three postpartum patients with PD, detailing the improvement in their symptoms.

**Case presentation:**

All patients in this study were married, in their thirties, and diagnosed using the Mini-International Neuropsychiatric Interview as having PD with agoraphobia. The Panic Disorder Severity Scale (PDSS) was used to evaluate patients’ panic symptoms and their severity. All patients received a total of 16 weekly 50-min sessions of CBT, and all completed the treatment. All patients were exceedingly preoccupied with the perception that a “mother must protect her child,” which reinforced the fear that “the continuation of their perinatal symptoms would prevent them from rearing their children”. After treatment, all participants’ panic symptoms were found to have decreased according to the PDSS, and two no longer met clinical criteria: Chihiro’s score changed from 13 to 3, Beth’s PDSS score at baseline from 22 to 6, and Tammy’s score changed from 7 to 1.

**Conclusions:**

CBT provides a therapeutic effect and is a feasible method for treating postpartum PD. It is important that therapists prescribe tasks that patients can perform collaboratively with their children.

**Electronic supplementary material:**

The online version of this article (10.1186/s40359-019-0330-z) contains supplementary material, which is available to authorized users.

## Background

### Postpartum women’s mental heath

In the field of women’s health, anxiety and depression symptoms are common in the postpartum period [1]. A recent literature review reported that generalized anxiety disorder, PD, obsessive compulsive disorder (OCD), and post-traumatic stress disorder are frequently diagnosed in postpartum women [2]. Specifically, the prevalence of clinically significant anxiety and depression, which is the most common mental condition during the postpartum period, has been observed at a rate of 10–20% in developed countries and approximately 30% in developing countries [3]. Also, the prevalence rates of PD in the general population range from 1.5 to 3.5% [4]; meanwhile, although using a small sample, a previous study showed that 11% of postpartum women have PD [5]. Thus, PD is more common in postpartum women than in the general population. PD is characterized by both recurrent and unexpected panic attacks, with at least one of the attacks having been followed by 1 month (or more) of one (or more) of the following: (a) persistent concern about having additional attacks; (b) worry about the implications of the attack or its consequences (e.g., losing control, having a heart attack, “going crazy”); (c) a significant change in behavior related to the attacks. PD is often (but not always) diagnosed alongside agoraphobia [4]. Untreated anxiety can have negative long-term consequences for both mother and child [6, 7]. Hence, it is importance to improve of symptoms by evidenced-based interventions. In the National Institute for Health and Care Excellence (NICE) guidelines, selective serotonin reuptake inhibitors (SSRIs) and Cognitive Behavioral Therapy (CBT), which have established effectiveness, are recommended as primary options for the treatment of PD [8]. However, SSRIs can cause adverse effects for fetus and infants and, thus, postpartum women are reluctant to take them [9]; regular exposure to SSRIs in the uterus is related to postnatal mental-health problems and an increased risk of fatal heart failure (hazard ratio: 1.17–1.38) [10, 11].

### CBT for PD

The CBT model for treating PD indicates that patients with PD can misinterpret normal physical sensations such as increased breathing, palpitations, and dizziness, and this can lead to panic attacks [12]. The CBT model seeks to help patients with PD understand that their internal physical sensations are normal, pursuing this outcome through behavioral experiments; for example, causing excessive breathing by asking the patient to run, or spinning the patient on a chair to make them dizzy. In Japan, we already reported feasibility of CBT for adult patients with PD by 2 single arm trials [13, 14]; We also confirmed a significant reduction of PD symptoms reporting a 60–80% improvement rate.

The use of CBT to address PD has been consistently found to be effective by meta-analysis including randomized controlled trials (RSTs) [15]. However, the effects of CBT are understudied. CBT’s previous research on perinatal depression and on effectiveness of psychotherapy for non-perinatal adult PD by the rigid systematic review has important implications for perinatal PD [2, 16]. The recent review by meta-analysis including 20 RCTs, including 3623 women, show that CBT as psychotherapy significant improved depression symptoms [16]. In addition, the review suggested that the intervention group had a lot of cured women than the control group that treatment as usual at almost of RCTs: Short term Odds Ratio: 6.57; Long term Odds Ratio: 2.00). In other words, CBT improve perinatal depression twice to sextuple as much as usual care. Although CBT models for depression and PD have different the hypotheses for maintenance of mood or anxiety, Intervention by CBT have targets of cognitions and behaviors in common. Therefore, CBT may be able to reduce panic symptoms as well as depression for perinatal PD. In addition, since literary prior research was in the Western culture area [2], it is important to consider CBT for perinatal PD in Eastern Asia as Japan.

### Responsibility in the postpartum patient with PD

A previous case series reported a mother who had become concerned that her child was isolated from the community and felt disappointed in herself [17]. The women experienced distressing symptoms, such as chest pain, palpitations, shortness of breath, dizziness, tightening of throat, blurry vision, amplified sounds, and tingling in extremities. They could not leave their homes, worrying about bad influence on their children. This mother’s suffering can be interpreted as a response to her love for her child reported that participants expressed feelings of guilt, avoidance, distancing and were completely distressed and overwhelmed by the responsibilities of motherhood.

A sense of responsibility for child care can promote excessive control of perinatal physiological and healthy responses. Obsessive compulsive disorder is a disease that strives excessively to fulfill one’s own sense of responsibility for things that can’t be originally controlled. The leading cognitive models of OCD posited that “inflated responsibility” beliefs play as a vulnerability and maintenance cognitive factor for obsessional thinking [18, 19]. Previous study by random-effect meta analyses included twenty-two studies (*n* = 8541, 48 effect sizes overall) suggested that “inflated responsibility beliefs may be associated also with symptoms of different forms of psychopathology other than OCD, specifically anxiety disorders. A possible explanation could be that responsibility beliefs play as a transdiagnostic cognitive factor for both OCD and anxiety disorders [20]”. Hence, it would probably be necessary to implement CBT to help such mothers recognize their responsibilities as mothers and the relationships they should have with their children. Additionally, postpartum PD can be caused by catastrophically misunderstanding physiological responses, because women can feel fear as a result of sensing abnormal respiration, dizziness, and changes in body temperature. Therefore, psychological education on the physiology of pregnancy may also be therapeutically important.

### Objective of this study

The objective of this study was to investigate the adaptability of CBT for postpartum PD of Japanese patients. Here, we present the results from clinical practice about postpartum PD of three patients. All patients had inflated responsibility for anxiety symptoms and physical sensation. In the current study, we focus on the CBT model of postpartum PD. We performed a retrospective study by three case series to assess the efficacy and feasibility of our CBT model for adult PD [21].

## Case presentation

### Participants

Participants were three women aged 36 to 38 years who met DSM-IV-TR criteria for PD [4]. Two patients had been referred by the obstetrics-gynecology to our psychiatry unit in Teikyo University Chiba Medical Center to treat their anxiety. Another patient had been referred by the psychiatry to our CBT center in Chiba University to improve her PD more. To distinguish among the three women in this manuscript, they have been assigned the fictitious names of “Chihiro,” “Beth,” and “Tammy.” Table 1 shows the patients’ characteristics: all participants have two infants. Range of children’ age was 0 to 4. Each patient had a supportive family and husband to whom each had been married for at least 7 years. Only Beth had lived in northern Europe, returned hometown to give birth her second child. Once her PD had improved, Beth planned to return to the country where her husband worked. These patients showed an increase in panic-related symptoms during the postpartum period and received CBT within 6 months of childbirth.

**Table 1:** Characteristics of the three patients

	Chihiro	Beth	Tammy
Age (in years)	36	38	36
Marital status	married	married	married
Education level	junior college	university	high school
Number of children	2	2	2
First child	4 years old girl	2 years old boy	6 years old girl
Second child	2 months old boy	3 months old boy	2 years old boy
DSM-IV-TR diagnosis	PD with agoraphobia	PD with agoraphobia	PD with agoraphobia
Pharmacotherapy	Sertraline 50 mg	Lorazepam 0.5 mg	Ethyl-loflazepate 1.0 mg

*DSM-IV-TR* Diagnostic and Statistical Manual of Mental Disorders, 4th edition, text revision

*PD* Panic disorder

In the assessment session before CBT, first author conducted interviews using the Mini-International Neuropsychiatric Interview (M. I. N. I.) [22–24], all patients exhibited sufficient criteria for PD diagnosis. Further, according to standard practice, the severity of their PD was measured using the Panic Disorder Severity Scale (PDSS) [25, 26]; Panic symptom severity levels ranged from mild to severe. The PDSS scores are shown in Table 2. None of the participants had previously received any cognitive behavioral intervention. Chihiro and Beth are reluctant to take regular medications, instead preferring to receive exclusively CBT introduced by their doctor (Second author: Sato K). Tammy already received pharmacotherapy and showed an improvement in symptoms. However, she hoped to receive CBT to further reduce the remaining panic symptoms. Tammy was also introduced to this psychotherapy by her doctor.

**Table 2:** Outcomes at pre-, middle-, and post-CBT

Patient	Scale	First session	8th session	16th session
Chihiro				
	PDSS	13	7	3
	GAD-7	10	7	5
	PHQ-9	7	6	7
Beth				
	PDSS	22	16	6
	GAD-7	10	8	4
	PHQ-9	6	5	4
Tammy				
	PDSS	7	3	1
	GAD-7	3	3	2
	PHQ-9	2	1	2

*PDSS* Panic Disorder Severity Scale

*GAD-7* Generalized Anxiety Disorder-7

*PHQ-9* Patient Health Questionnaire-9

### Measures

To provide data on the effectiveness of the therapy, participants completed assessment surveys reporting panic, general anxiety, and mood during daily life in the first, middle, and final CBT sessions. The primary outcome was measured using the PDSS [25, 26]. The PDSS is a 5-point Likert-type scale ranging from 0 (not severe) to 4 (severe). The PDSS is a seven-item clinical interview rating scale that assesses the core features of PD. The seven items include (1.) the frequency of panic attacks and episodes with limited episodes, (2.) panic attacks and LSE distress, (3.) anticipatory anxieties, (4.) avoidance, (5.) fear and avoidance of panic-related sensations, (6.)occupational dysfunction, and (7.) social dysfunction. Evaluation using this scale takes 10–15 min. As a preliminary analysis, the PDSS survey shows useful psychopathological characteristics [27]. To measure general anxiety and mood, generalized anxiety symptoms and depressive symptoms were measured by the patients’ therapists using the Generalized Anxiety Disorder-7 (GAD-7) scale and the Patient Health Questionnaire-9 (PHQ-9), respectively [28–30]. The GAD-7 was designed to identify probable cases of generalized anxiety disorder and to assess symptom severity. The items featured on the GAD-7 describe the most prominent diagnostic features of the DSM-IV diagnostic criteria A, B, and C for generalized anxiety disorder [4]. In accordance with the GAD-7, subjects are asked how often, during the last 2 weeks, they have been bothered by each of the seven core symptoms of generalized anxiety disorder: (a) feeling nervous, anxious, or on edge; (b) uncontrollable worrying; (c) worrying too much; (d) trouble relaxing; (e) restlessness; (f) feeling annoyed or irritable; (g) feeling afraid as if something awful might happen. The PHQ-9 consists of nine items to assess the presence of the nine diagnostic criteria for major depression according to DSM-IV [4]. The PHQ-9 evaluates the presence of the following symptoms over the previous two-week period: (a) depressed mood, (b) anhedonia, (c) sleep problems, (d) feelings of tiredness, (e) changes in appetite or weight, (f) feelings of guilt or worthlessness, (g) difficulty concentrating, (h) feelings of sluggishness or worry, and (i) suicidal ideation. Items on both the GAD-7 and PHQ-9 are answered on a four-point Likert scale from 0 to 3 as follows: 0 (never), 1 (several days), 2 (more than half of the days), and 3 (most days).

### Therapist and supervisor

As the aim of this case series was to learn how to adapt CBT for postpartum PD, it was essential that the therapy and supervision be conducted by individuals fully trained in adult PD. All sessions were delivered by Matsumoto K., an experienced clinical psychologist who had been trained in CBT for anxiety disorder during a clinical placement at the Center for Cognitive Behavioral Therapy, Chiba University Graduate School of Medicine, Japan. Matsumoto K had been provided weekly individual face-to-face supervision by Shimizu E who developed the CBT model for PD.

### Treatment

Therapy was delivered in accordance with the standard adult protocol (i.e., 16 individual 50-min sessions). The CBT model is administered once per session, with the exception of the “behavior experiments,” which were repeated five times in a row. After each session, Matsumoto K carefully reviewed the session and discussed with Shimizu E the effect panic seemed to have on the patient’s cognition and behaviors, along with plans for the next session. All patients completed 16 sessions of the CBT model [21].

**Fig. 1:**
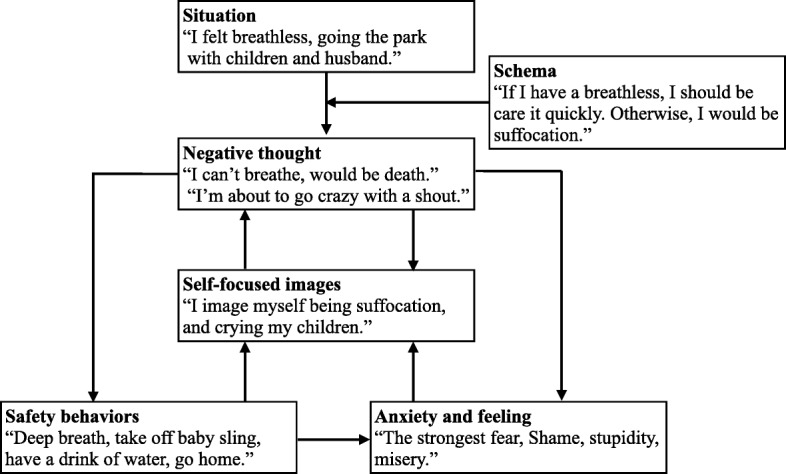
Patient 1’s case-formulation

The following treatment components were conducted with the aid of worksheets delivered during the session and also as homework:

1. Assessment and goal setting: The therapist performed hearing of mental health history, evaluated severity of panic symptoms by PDSS, and provided feedback to patients. Through this, the patients could gain an understanding of themselves (including their symptomology), establish therapeutic goals, and increase their focus on addressing PD by CBT. In Session 1, the patients were asked, as homework, to think of the thoughts and behaviors related to a feeling of panic that they experience or perform in their daily lives.
2. Psycho-education regarding the CBT model: The application of Seki and Shimizu’s CBT model was determined collaboratively with the postpartum women, based on their own thoughts, images, and safety-behaviors [21]. The model used paper- or digital-based visual aids created by the therapist to help the patients easily understand their panic. In the course of relaxation, the therapist relieved the patients’ physical tension by instructing them in normal, rhythmic breathing; sometimes, the therapist demonstrated this by performing the behaviors him/herself.
3. Case formulation: Fig. 1 depicts Chihiro’s model. The relationship among the three elements of 1) attention to internal information from body sensations, 2) critical misinterpretation, and 3) safety behavior combined to invariably cause the patients to maintain their panic levels [31]. The therapist illustrated this vicious cycle of panic to the patients using a visual aid.
4. Safety behaviors: Therapist helped the patients identified safety behaviors. To examine the functions of the patients’ safety behaviors, in Session 4, two ways of role-playing (both with and without safety behaviors) were demonstrated. In the first trial, the patients were asked to focus their attention on themselves and think of a panic attack, while also performing their habitual safety behaviors (including maintaining an internal attention condition). In the second trial, they were encouraged to focus externally, not to perform safety behaviors, and instead to involve themselves in their situation (external attention condition). Typically, patients with PD discover that the habitual safety behaviors by which they perceive internal physical sensations (being self-focused and evaluative) makes them feel more anxious. It is important that patients empirically recognize their unconscious safety behaviors and maintain an awareness of their internal and external attention conditions. As homework, patients were recommended to repeat the two approaches on a daily basis.
5. Re-constructing the catastrophic self-image associated with internal physical sensations: During panic-related episodes, visual images that caused pain may also occur, along with symptoms of post-traumatic stress disorder [32], and these can last longer than the instigating thought. If the patient converts the visual image into a linguistic format (i.e., through speaking or writing it), it disappears more quickly [33]. Based on the negative emotions arising from critically exaggerated interpretations of physical sensations, patients with PD overestimate the true threat and create catastrophic images [34]. In Session 5, in order to establish an identification of images, the patients first, with their eyes closed), were asked to express the most catastrophic image that, for them, can cause a panic attack involving symptoms such as palpitations and hyperventilation (e.g., dying on the street because an ambulance does not arrive). Next, the meaning of the image was discussed, such as through considering evidence and falsifications, intelligently reconstructing the meaning of the image in order to increase the patients’ confidence. Finally, the therapist encouraged the patients to create positive images, and discussed with the patients the relationship between the safety behaviors and the image.
6. Attention-shift training: Patients with PD tend to excessively focus their attention on internal physical sensations (palpitations, hyperventilation, dizziness, etc.) [35], and become hypersensitive to unusual sensations [36], thus making, anxiety symptoms more likely to occur. Therefore, it is necessary to direct attention to external, non-physical sensations (sounds, colors, figures). In addition, for patients who try to remain focused on external attention to avoid the fear of internal feelings (a safety behavior), the goal is to be able to freely and flexibly shift between internal and external attention.
7. Behavioral experiments regarding catastrophic beliefs: It was necessary to conduct behavioral experiments across multiple sessions, including interceptive exposure and in vivo exposure. Hence, we collaboratively devised experiments to examine the patients’ catastrophic beliefs regarding their physical sensations. During the experiments, in order to collect new information about their panic, patients were encouraged to stop performing their safety behaviors and to focus their attention externally. This was designed to help patients realize that the feared catastrophic outcome is less likely to occur than they originally believed (see Additional file 1 for examples with the postpartum patients). Behavioral experiment was administered in session 7 to session 11, which was repeated five times in a row. Patients were recommended for exposure to select methods based on their diagnosed level agoraphobia, based on the anxiety hierarchy chart.
8. Re-scripting early panic memories associated with negative images: Patients with anxiety disorder are more likely to recall content relating to threats [37], which makes them hypersensitive to stimuli related to threats and more likely to retain such stimuli in their memory [38]. Therefore, after the first intense panic attack, some patients experience the event as a trauma. As part of this CBT intervention, after identifying the traumatic memories, the patients addressed these memories using techniques and empathic words learned through CBT. This process overwrites the implications of the event and attributes a more positive meaning to a panic attack. The patients had reduced mental defeat and increased cognitive flexibility, through “imagery re-scripting,” so that they came to be able to manage the meaning of images and memories associated with the first panic attack situation [39].
9. Modifying pre- and post-event processing: By reflecting on behaviors during and results after a panic attack, a patient tries to confirm the correctness of their safety behaviors as ritual actions. As a result, they develop increased confidence in false beliefs. Consequently, patients with PD must cease engaging in such pre- and post-event processing. By writing down their ruminations and recording the specific nature of their worry surrounding an event and analyzing the relative merits and demerits of such thoughts, patients can usually choose not to engage in such habits in future.
10. Opinion survey regarding others’ evaluations of a catastrophic situation: Even if the worst situation (such as hyperventilation and fainting) occurs, patients need to be aware that others will not evaluate them as negatively as they believe. To assess the criteria and viewpoints of others, public opinion surveys were conducted.
11. Schema work: Negative nonfunctional beliefs/assumptions (schema) were identified in this session. For example, an extreme cautionary warning is: “I must always be careful about chest palpitations.” For this, the conditional belief is: “I could die unless I carefully monitor myself for chest palpitations.” Meanwhile, the unconditional belief is: “No matter what I do, I will suddenly die.” To address this, patients were asked to create positive functional beliefs/assumptions instead of relying on schema, and to write them on cards so that they could be referred to at any time. For example, “If I feel heart palpitations, it is not an actual heart attack; for example, I can still walk.” As homework, patients were asked to recite the contents of the cards they created every day and record evidence of positive emotions supporting the new belief.
12. Preventing relapse: The therapist listened to the skills and knowledge the patients had acquired through their treatment and gave them feedback regarding their demonstrated level of awareness. In addition, to generalize what the patients had learned from previous PD-related episodes, the therapist held collaborative discussions with the patients.

#### Patient 1 – Chihiro

Chihiro was 36 years old at the first session. She graduated from college and worked full-time as a retailer for a decade. She then married in her early thirties and retired from full-time work, taking a part-time position as a clerk. After 2 years, Chihiro became pregnant; she consequently retired from her career and began living a happy life at home. Chihiro’s decision to retire was an easy one; caring for her children was her priority. Four years later, she had a second child, for whom she had hoped and planned. For this second pregnancy, Chihiro needed to be hospitalized and underwent a cesarean section. Immediately after hospitalization, Chihiro began to experience symptoms of anxiety, such as feelings of compression, stuffiness, cold sweats, and a strong fear that she was “about to scream and lose control.” However, as the birth of her child was imminent, the nurse asked Chihiro to “please stay in your room,” so she remained in her hospital room, experiencing repeated panic attacks. Chihiro gave birth without complications, and she felt relief upon meeting the new member of her family. Unfortunately, Chihiro’s panic attacks did not cease after discharge, so she visited a psychiatrist for help. Chihiro thought, “if I keep having panic-attack symptoms, I will not be able to be a good mother because I won’t be able to do things such as take the children to the park.” The second author, who became Chihiro’s attending psychiatrist, diagnosed her with PD and agoraphobia using DSM-IV-TR criteria [4]. At the time, because Chihiro was lactating (as it was 2 months after childbirth), CBT was initially administered without medicinal intervention, but Chihiro suffered a strong panic attack after the fifth session. After this, her doctor prescribed her an SSRI (25 mg of sertraline). From the seventh session, the level of sertraline was increased to 50 mg, and this continued until the final CBT session.

In the first CBT session, we noticed that Chihiro constantly focused her attention on her throat. Such a habit made her hypersensitive to throat discomfort. Conversely, Chihiro also held the belief that “if I pay too much attention to my throat, I will suffer a panic attack.” To verify Chihiro’s catastrophic beliefs regarding physical sensations, the therapist and Chihiro undertook behavioral experiments by performing activities that caused her to feel fearful (e.g, taking the elevator, going to the cinema and sitting in the middle of the venue, exercising, going out with her children), without performing safety behaviors; in one task, in order to simulate a breathless experience Chihiro was asked to climb stairs quickly, which she performed with the therapist in the hospital, as follow:

*KM: If your speculation is correct (if you don't cope with it right away you will asphyxiate), you must always get a panic attack when you feel of dyspnea, right?*

*Chihiro: I agree. Even with this care, I always feel like my throat is full.*

*KM: Another way of thinking is explained in the CBT model of PD. It is a hypothesis that you are catastrophically interpreting the physical sensations and focusing attention on internal information has made it easier to notice the unpleasant physical sensations.*

*Chihiro: That might be true. It is too difficult for me to protect my children if panic attach occurs to me, so that I always be concerning about the condition of my throat after a panic attack has occurred.*

*KM: It is a natural thing to happen to a healthy person to feel tightness when exercising or doing activities. Chihiro's breathlessness may also be healthy and harmless. Dare, Could you do activities that feel of dyspnea and reassess the danger? First task is climbing the stairs of this hospital with me, from the first floor to the ninth floor.*

*Chihiro: Well…. OK, I will try.*

*KM: Brave decision. Let's challenge now!*

To expose Chihiro to compression and breathlessness from physical internal sensations, the following homework tasks based on behavioral experiments were established: to expose internal sensations such as breathless or compression, she would exercise by climbing up and down a low platform for 10 min, wrap threads for cooking around her wrists, and use a hug string when hugging her baby. Through the series of behavioral experiments, Chihiro learned that her fear of physical sensations was unfounded. After performing the experiments, Chihiro found it easier to go out with her children. Finally, she was able to bring her children to a show for children, which required her to get on a limited express train whose doors remain sealed for over 10 min after the train has departed. She was glad that her children, especially the eldest son, seemed very happy to be able to go out with their mother. Chihiro’s PDSS scores for panic symptoms fell from 13 pre-treatment to 3 post-treatment: GAD-7 scores from 10 to 5; PHQ-9 scores from 7 to 7 (see details in Table 3).

**Table 3:** Patients’ performance in each session

The protocol	Patient 1 (Chihiro)	Patient 2 (Beth)	Patient (Tammy)
1) Assessment and goal setting (Session 1)	Symptoms associated with panic: Asphyxia, palpitation, shortness of breath, strong fear Catastrophic interpretation: “Being stifled and suffocating” Inflated responsibility: “I am a mother, but I can’t protect my child if a panic attack occurs. I must make an effort to absolutely not cause a panic attack.” Goals: Learning to cope with breathlessness and gradually becoming free from it. Being able to ride in a vehicle without problems and finally living without worrying about panic attacks.	Symptoms associated with panic: Palpitations, fluttering, dizziness, hand tremors, feeling faint. Catastrophic interpretation: “If I feel my heart beat rapidly, I will fall down soon.” Inflated responsibility: “If something happens to me, my husband’s job will suffer, and he will be negatively evaluated by his boss/colleagues. My panic attack will have a bad influence on the child’s rearing and education. Hence, I always have to immediately recognize and deal with the physical discomfort.” Goals: Visiting the office she worked and her friend’s house with her children by train. Getting a haircut by a hairdresser for the first time in a year. Overcoming the daily breathlessness, dizzy, and worrying about the next panic attack.	Symptoms associated with panic: Tightness, heat, sweating, palpitation, fear of insanity. Catastrophic interpretation: “If I recognize my physical symptoms, I will feel suffocated, my body will heat up, and I will lose control over myself.” Inflated responsibility: “If I feel the beginning of a panic attack, I cannot play with or take my children anywhere. I must be in control of my physical symptoms. Goals: Playing with children and living everyday life without worrying about panic attacks.
2) Phyco-education (Session 2)	First, the therapist told them: “Pregnancy is a period during which physiological, psychic, hormonal, and social changes take place, increasing the risk of psychiatric morbidity in this stage of a woman’s life [40].” “Some women experience pregnancy as a source of happiness, satisfaction, and self-fulfillment. Others experience a change in their mental health, such as the development of anxiety [41].” Second, the therapist introduced the concept of PD and the CBT model of PD [34]. All patients who listened to this information said that “PD fits my symptoms of anxiety or panic attack.”		
3) Case-formulation (Session 3)	See Fig. 1.	Situation: At a husband’s promotion party, standing next to her speaking husband Schema: I fall over feeling scared and dizzy Negative thought: I’m nervous; I’ll fall flat on this Self -focused image: If I fall down, I will be judged as a mentally weak person Anxiety: Palpitation, asphyxiation, wandering, heat, sweating Safety behaviors: Apply strength to your body not to fall down, grasp the husband’s arms firmly, say “OK.”	Situation: Enter the hairdresser’s alone and sit in a chair Schema: A panic attack occurs if you do not cope with breathlessness immediately Negative thought: Feeling suffocation will get worse Self-focused image: I’m struggling and suffering myself Anxiety: Asphyxiation, hace and neck feel flushed with heat, restless, fear Safety behaviors: Breathe deeply, drink water, go outside the store and take in deep breaths of the outside air
4) Safety behaviors (Session 4)	Wear loose clothes, refrain from exercise, always worry about the condition of the throat, and have her family accompany you when you go out.	Do not put a burden on the body as much as possible, do not go out as much as possible, use the Internet to immediately check any concern, as well as the necessary place to visit.	Do not take a hot bath, refrain from playing with children, do not lift children, refrain from going out, stop going out.
5) Re-constructing the self-image (Session 5)	Shoot me when I feel stuffy and watch it on the video.	When I feel dizziness or flutter, I stand in front of the mirror and observe realistic situation.	Watch the role play video when you are on the train.
6) Attention training (Session 6)	Visual: Counting the colors in the consultation room. Taste: Drinking tea and describing the taste. Hearing: Children’s voices and footprints. Tactile feeling: The feeling of holding a baby.	Visual: Counting the color of the counseling room, verbally describing the children’s appearance Taste: Drinking tea and explaining the taste	Visual: Look at the landscape Smell: Depict the smell Hearing: Explain the sounds you hear
7) Behavioral experiments (Sessions 7–11)	Interceptive exposure: Excessive Breathing; Stair dash from first floor to 9th at the hospital. Situations of in vivo exposure: Exposure to anxiety situations (nearby small parks, fast trains, road trips while sitting in the back seat of a car, and amusement parks).	Interceptive exposure: Breathing through a straw; Turn around on a swivel chair; Stair dash. Situations of in vivo exposure: A train at each station; Limited express trains; Shopping malls.	Interceptive exposure: having a shower on face; handstand; squat; walking hardly. Situations of in vivo exposure: Driving a car with children; Large parks in the suburbs; Express trains over more than one hour.
8) Re-scripting early panic memories (Session 12)	Intervened in panic attacks that occurred during hospitalization for second child delivery.	Intervened in a panic attack that occurred at her husband’s promotion party.	Intervened in the panic attack that occurred in the beauty salon after the miscarriage,
9) Modifying pre- and post-event processing (Session 13)	Don’t over-reserve and don’t take a break after it’s over.	go out without preparing too much, worrying about physical illness, or examining various things.	Don’t rehearse in my head, come back home, or consult husband.
10) Opinion survey (Session 14)	Chihiro noticed that even if she had a panic attack, surprisingly nobody would blame her or care.	Beth was worried when she had a panic attack in public.	Tammy, as expected, reaffirmed that people around her were tolerant of panic attacks.
11) Schema work (Session 15)	The catastrophic interpretation has been transformed into a safer one, saying, “Even if I get stuffy, I’ll be relieved.”	The catastrophic interpretation was corrected from “I will not fall even if I have a tightness.” to “If I go out, I will not feel tight because I will not be paying attention to it,” and “I can cope with tightness.”	The catastrophic interpretation has been corrected to be safe, “It will be manageable even if I feel stuffy, or sealed, or receiving emergency hospitalization, etc.”
12) Preventing relapse (Session 16)	Daily exposure to tightness and asphyxiation. If Chihiro can’t respond on her own, she must go to the hospital without hesitation.	If Beth can’t cope, she must go to the hospital. She must take her children to the park regularly even if she feels uneasy	If I cannot cope, I will go to the hospital. Play with children without hesitation. Having experience that the tightness and heat flushes daily by interceptive exposure.

In Chihiro’s case, the change in the her living environment seemed to cause the absence of a change in the PHQ-9 value from before and after treatment, despite marked improvement in panic symptoms. In Japan, there is customary for a postpartum daughter live for a while at her mother’s house. After a daughter has recovered from the fatigue of childbirth, she will return to her husband’s home. At the end of the treatment program, Chihiro was returning home with her children, and so she no longer had the support of her parents’ constant presence. Therefore, the relative increase in Chihiro’s nurturing role may explain the post-treatment PHQ-9 score (the same value recorded during pre-treatment).

#### Patient 2 – Beth

Beth was 38 years old at the first session. After graduating from university, Beth worked full-time as a general clerk for 15 years. Marrying in her mid-thirties, she soon gave birth to her first child at the age of thirty-six. On an otherwise normal day when Beth had been driving, she suffered a panic attack. She felt a sharp panic manifest in the form of a rapid heartbeat, breathlessness, and serious fear. Since then, Beth often experienced palpitations, difficulty breath, trembling, and high levels of fear, both in her car and at home. In the past, Beth had been treated with psychosomatic medicine, and further anti-anxiety medicine was prescribed (specifically ethyl loflazepate, as well as other medications whose details are unknown to the authors) to her to take regularly for 1 year, but it was ineffective. Further, due to circumstances regarding her husband, she relocated to a European country, and her treatment was temporarily suspended. Although Beth wanted to take walks with her child in the beautiful European townscape, she never did because she feared “if I feel dizzy and my breathing gets difficult, I may faint; and this will reflect badly on my husband and could have a negative effect on his career.”

Beth’s strongest supporter was her husband, who listened to her problems and anxieties every day. Beth was able to go out with her husband and baby every weekend, and this made her happy. Then, Beth was pleased to find that not long after moving to Europe, she had become pregnant with her second child. However, her anxieties about her panic attacks also increased. Approximately 10 months after moving abroad, Beth returned to Japan to give birth. After giving birth she felt unable to fulfill her responsibilities as a mother with respect to her baby’s care and education, saying “I can’t bring my child anywhere as it is, and I feel depressed all day if I do not go out.” As a result, she visited a psychiatric department for the purpose of curing her panic and anxiety. Using DSM-IV-TR criteria, Beth was diagnosed with PD with agoraphobia [4]. As Beth wanted to breastfeed her baby, she consulted with the attending psychiatrist carefully and decided to undergo individual CBT rather than pharmacologic therapy.

As part of the CBT, Beth performed role play that involved boarding a train. She was asked to notice her strong anxiety when she became worried and to pay attention to her respiration. Although she appropriately engaged in external attention during the session, she later reported that she was unable to do so during homework because her child’s “playing was very noisy, and I could not concentrate.” Consequently, we adjusted the task to flexibly accommodate and bring attention to the sounds of children playing as well as her physical sensations. Beth answered, “I will do it while breastfeeding,” and later reported that she had succeeded, stating that: “I became confident in my ability to flexibly manage my attention, and I was able to go to a hairdresser for the first time in a few months.” In a behavioral experiment, Beth sat on a rotating chair, and was spun around five times, making her dizzy. She then rushed up some stairs (with the therapist behind her as a safety precaution). Through this activity, Beth experienced heavy breathing and dizziness. Additionally, focusing on her internal physical sensation allowed her to develop the reasonable interpretation that “if I feel difficulty breathing, I will not succumb to a little dizziness.”

Beth also believed that “when a person feels dizziness or shortness of breath, because my husband’s boss or colleague and their wife will not respect a person whose family member has a PD, it reflects badly on my husband.” To help her understand that others would not make this evaluation, the therapist first confirmed that Beth herself did not negatively evaluate others based on their spouse’s physical symptoms as follows:

*KM: "If your friend raise panic attack in front of you, would you evaluate her or him as a bad mother/wife?"*

*Beth: "No at all."*

*KM: "Why do you evaluate yourself negatively?" “Is that reasonable?”*

*Beth: "Well, now that you say that, I may be too harsh for myself."*

Next, we performed an opinion survey with 10 people, affirming that, if a person collapses from a panic attack, none believed that “that person’s family can’t be trusted,” or “that person’s family can’t function.” The people recruited for the survey were Beth’s three family members (father, mother, and husband) and seven colleagues of the authors. These efforts allowed Beth to become aware that “people do not think critically about others in a negative way,” and somewhat relieved the anxiety that caused Beth’s panic attacks as follows:

*KM: "According to the questionnaire, no one answered that they could not trust anyone who had panic attacks. How would you interpret this result?"*

*Beth: "Unsurprisingly everyone was kind, they don't evaluate negatively. I found that I was the most critical of my own symptoms."*

In the final session, Beth told the therapist that “I would like to take my children to a beautiful national park near my residence in Europe,” to which she later returned. Her panic symptoms pre- and post-treatment changed from 22 to 6 on the PDSS scale, respectively. Her GAD-7 scores changed from 10 to 4, and PHQ-9 scores changed from 6 to 4 (see details at Table 3).

#### Patient 3 – Tammy

Tammy was 36 years old while first session. Tammy is the mother raising two children. After graduating from high school, Tammy began working full-time. Then, at the age of 29, Tammy got married. After her marriage, she took a part-time job in sales, which she retained until she gave birth to her first child. Later, she gave birth to a second child, a daughter. Tammy wanted to raise more children so, at the age of 36, she planned to become pregnant again. Soon after becoming pregnant, Tammy was sitting in her car on a sunny summer day waiting for a traffic signal when she suddenly suffered a panic attack: “my head felt hot, I could not breathe; the panic made me so scared that I returned home.” While delighting in her pregnancy, a sufficiently stressful situation to cause another panic attack did not arise for some time. However, Tammy suffered a miscarriage and her emotional state changed. She suffered a panic attack when visiting to a familiar beauty salon by herself. The feeling of heat when hot water was poured onto her hair made Tammy afraid: “my head was hot, I became afraid! I could not run away from there, as I was fixed into a chair.” Leaving the beauty salon allowed her to calm herself; however, anticipating further panic attacks, she returned home without having her hair cut.

A month later, Tammy continued to experience troubles in daily life, such as sudden panic attacks and fear when going out. As a result, Tammy went to a psychiatrist who was recommended to her by her family physician. Using DSM-IV-TR criteria, the psychiatrist diagnosed her with PD and agoraphobia [4], prescribed 1.0 mg of ethyl loflazepate. The effect of this pharmacotherapy was remarkable, as Tammy stated that “my daily life has become much easier,” but anticipatory anxiety remained. Therefore, Tammy decided to undergo CBT, a decision she made by consulting with her psychiatrist 4 months after beginning pharmacotherapy. Considering Tammy had responsibilities in raising her children, we agreed to conduct CBT via video-conferencing in order to ensure that treatment could be provided at a regular frequency through 50-min weekly sessions.

After explaining the CBT model for PD and formulating a protocol based on Tammy’s panic symptoms, activities to simulate these symptoms were affected. Tammy found that efforts to focus on the dryness in her throat and breathing kept her constantly aware of those sensations, and that her safety behaviors in this regard were taking deep breaths or having a drink of water. Through role playing, we verified that her safety behaviors were maintaining her anxiety. As a result, Tammy agreed to engage in daily life without performing safety behaviors. For example, Tammy often noticed her breathing when lying down and observing her child sleeping (in Japanese culture, mothers lying beside pre-school children is considered to be a good child-rearing method). In recognition of this behavior, tasks in behavioral experiments involving exposure to internal senses were established, including breathing through a straw, excessive breathing, putting warm water on her face, and submerging her face in hot water while in the bath. Every task served to reinforce a new belief that breathlessness is not related to panic attacks. Tammy later reported that: “I can start house keep, but I cannot carry on doing them.” Therapist found that she had traumatic memory about panic attack. She changed the meaning of trauma memory to a safer thing: from “I suffer from panic symptoms that never recover to helpless” to “I know how to cope with panic and the panic attack will vanish over time.” In intervention for her traumatic memory, She advised past-herself that “I know you were afraid. Don’t worry, paying attention to bodily sensations and uneasy thoughts makes you uncomfortable, so you should read some magazines or engage in some other behaviors that distract you.”

Tammy used empathic words to give advice to her past self. Additionally, Tammy was fearful of getting on a train, but, by regularly performing an activity involving boarding a bus or train, she was finally able to board a bullet train and remain on board for over an hour. The severity levels of her pre- and post-treatment panic symptoms decreased from 7 to 1, respectively, as measured by the PDSS. GAD-7 scores changed from 3 to 2 and PHQ-9 scores changed from 2 to 2 (see details in Table 2).

## Discussion & Conclusion

The present case series concerned the efficacious application of CBT to treat three women with PD related to reproductive events. Specifically, it involved the use of the Seki and Shimizu CBT model [21], which has been demonstrated to be effective for adult patients with PD; the model comprises 16 weekly 50-min sessions. In this case series, all patients performed all aspects of CBT and consequently showed improvements in their panic symptoms. They all experienced panic attacks during the postpartum period and had safety behaviors that reinforced their excessive anxiety regarding their physical conditions and physical sensations. These feelings seemed to evolve from their viewpoints regarding the duty of a mother to raise her children. It is possible that women after childbirth judge normal autonomic nervous system responses, such as anxiety, shortness of breath, palpitations, etc., to be a great threat to her own and her children’s lives, and consequently pay excessive attention to their physical sensations and safety behaviors [42]. All patients were socially required to play a role as a mother, and they were hoping to fill the role one their-self. Such physiological and social contexts during pregnancy and after birth may affect all elements of the CBT model vicious cycle, such as occurrence of physical symptoms, schema, automatic thinking, self-image, safety behavior, and anxiety symptoms. We suggest that it is important for postpartum PD to intake that context in each session, since CBT tasks that are directly related to protecting and caring for children were more performed than others.

In a previous study, it was indicated that beliefs concerning inflated responsibility for causing or preventing harm to oneself or others plays a critical role in the maintenance of compulsive checking behaviors and other form of obsessive behavior characteristic of OCD [43]. The findings of the present case series accord with those of previous studies that have shown that attitudes toward child rearing and responsibility as a mother influence the manifestation of symptoms of PD. Therefore, in order to prevent PD and unnecessary anxiety in this population, it is suggested that during the postpartum period women be provided with psycho-education on perinatal physiology and mental health. Also, a belief that closely resembles OCD was observed in the three patients in the present study: “inflated responsibility” [44], this involves the belief that a threat can be avoided depending on one’s own efforts, resulting in the threat being excessively estimated. The definition of “responsibility” in obsessive-compulsive disorder is that there is an important force that can cause or prevent the consequences one fears [45]. With regard to the beliefs that affect the onset of mental illness, PD is characterized by interpreting the physical sensation catastrophically [12]. We found that perinatal PD patients often have responsibility which prompts fear or anxiety concerning their ability to take care of the baby. As a result of this observation, we suggest that catastrophic interpretation of responsibility be used to predict the onset of perinatal PD. The finding among Japanese PD patients in the perinatal period support the previous findings that perception of responsibility is a risk factor which can be used in cross-diagnosis of anxiety disorder and obsessive-compulsive disorder [19].

In CBT, it is important to set tasks that can improve patients’ standard of living, and to have the patients continue to perform these tasks in their own living environments [46, 47]. In these case studies, all patients were instructed to bring their children to the city park or the shopping mall, and, upon doing so, they consequently reported a strong sense of accomplishment. It is thought that setting tasks that mothers can perform with their children is important for motivation, and such tasks also have high levels of utility in everyday life. Considering that mothers seem to fall into a vicious cycle of panic as a result of their obligations to protect their children and their sense of responsibility regarding child rearing, it is natural that tasks involving the role of a mother being used as motivation. The findings of our report further support that it is critical to establish behavior al experiments and homework tasks that involve children, particularly for mothers with postpartum PD.

When raising multiple children, it may be difficult to visit a hospital frequently. In the series of these cases, Chihiro and Beth received support from their parents, who took care of the children during their daughters’ CBT sessions. However, Tammy did not receive support from her parents, so it was difficult for her to visit our facility at least once a month; consequently, she underwent CBT via video-conferencing. Two recent systematic reviews, through meta-analyses, suggested that Internet-based CBT (ICBT) were significantly effective in reducing anxiety from which a patient with anxiety disorder suffered [48, 49]. The effectiveness of ICBT for OCD during pregnancy has been reported in a trial involving RCT design [50], several studies reported the effectiveness on ICBT for PD [51–55]. To examine the feasibility of ICBT for adult patients with PD, we conducted a clinical trial using a videoconference system, as a result of which the symptoms of panic and agoraphobia were significantly reduced [13]. Hence, ICBT, which is composed of web-based treatment programs, involves minimal guidance, allows for correspondence via e-mail, telephone, or video-conferencing, and allows therapists to deliver effective treatment to patients at home on a flexible schedule. Such ICBT approach promises to be useful for pregnant women with PD.

This study is limited in several ways. First, this study is observed retrospectively, it cannot be explained with a causal relationship. To prove the causation of postpartum PD in pregnant women by their strength of their sense of responsibility, future research has to investigate the risk of perinatal PD in a cohort study. The second limitation relates to cultural differences. This study is the first in the world to report the efficacy of the application of the CBT model to Japanese women suffering from postpartum PD, and, as such, should not be extrapolated to the other populations without further research on those specific populations. Since similar studies have not yet been conducted in other countries, this paper cannot predict the efficacy of the CBT model to non-Japanese patients. The final limitation is the failure to ascertain conclusively the effectiveness of the CBT model for perinatal PD. This study cannot claim certain effectiveness, because the series included only three case studies. In order to examine the efficacy of the CBT model for postpartum PD in the future, it is necessary to conduct clinical trials designed to include random controls.

In the present case studies, the patients showed improvements regarding their panic symptoms pre- and post-intervention and did not report any adverse events. Our results show the viability of CBT for patients with PD after childbirth.

## Additional file

Additional file 1: Behavioral-experiments. (DOCX 16 kb)

## Data Availability

Not applicable.

## Abbreviations

CBT: Cognitive behavioral therapy
GAD-7: Generalized Anxiety Disorder-7
ICBT: Internet-based cognitive behavioral therapy
MINI: Mini-International Neuropsychiatric Interview
NICE: National Institute for Health and Care Excellence
OCD: Obsessive compulsive disorder
PD: Panic disorder
PDSS: Panic Disorder Severity Scale
PHQ-9: Patient Health Questionnaire-9
RCTs: Randomized controlled trials
SSRI: Selective serotonin reuptake inhibitors
