# Supplementary material for: Cognitive behavioral therapy for postpartum panic disorder: a case series
Source: BMC Psychol. 2019 Aug 22;7:53. doi: 10.1186/s40359-019-0330-z (PMC6704562; doi:10.1186/s40359-019-0330-z)
Supplement: Supplementary file 1 — Behavioral-experiments. (DOCX 16 kb) [file 40359_2019_330_MOESM1_ESM.docx]

**Additional file 1. Behavioral experiments for catastrophic beliefs relating to physical sensations**

**Example behavioral experiment with Chihiro**

*Target physical sensations:* Breathlessness, choking sensation, and heart palpitations

*Predictor:* “When I experience breathlessness or a choking sensation, I must address it immediately. Otherwise, I could get hyperpnea” [belief rating: 65%].

Experiment: “Without attempting to address breathing state, climb the stairs at the hospital from the first floor to the tenth floor, with the therapist.”

Outcome: “My breathing felt the same as usual, but I could climb the stairs to the end without hyperpnea. My breath returned to normal after a while.”

What I learned: “Breathlessness is not evidence that I have hyperpnea. To improve my PD, I should practice this experiment.”

New level of belief in original prediction: 40%

**Example behavioral experiment with Beth**

Target physical sensation*:* Dizziness

*Predictor:* “When I feel weakness and dizziness I will fall down if I try to stand up” [belief rating: 60%].

*Experiment:* Sit on a rotating chair and quickly spin around five times, and then stand up.

*Outcome:* “I felt strong dizziness, but when I stood up I did not fall down. I did not have to use a lot of effort in my lower limbs to balance myself.”

What I learned: ”Because, I learned that if I feel dizzy I will not necessarily fall down, I feel more confident about going out.”

New level of belief in original prediction: 20%
